# Editorial for the Special Issue “Polymeric and Polymer Nanocomposite Materials for Photonic Applications”

**DOI:** 10.3390/polym12123036

**Published:** 2020-12-18

**Authors:** Sergi Gallego, Yasuo Tomita

**Affiliations:** 1 Department of Physics, System Engineering and Signal Theory, Universitat d’Alacant, E03080 Alicante, Spain; 2 Department of Engineering Science, University of Electro-Communications, Tokyo 182-8585, Japan; ytomita@uec.ac.jp

Polymer nanocomposites are designed and engineered on a nanometer scale with versatile applications including optics and photonics. Initially, photopolymers were used in areas such as reflecting flat-panel displays, optical interconnects, holographic optical elements, diffractive lenses, optical data storage, solar concentrators, wearable/see-through displays, sensors, etc. Depending on the application, the chemical composition of the photopolymer should be optimized. In this sense, the introduction of nano-compounds into the polymeric structures can strongly modify their properties and open new possibilities. 

This Special Issue highlights the continuous growth and advancement of photopolymers and nanocomposites used for optics and photonics. In order to maintain successful growth and diversity in these applications, a better understanding of photopolymers as optical recording materials is necessary for more innovative device applications.

During the last two decades, different photopolymerizable nano-compounds were introduced and developed to modify polymer properties. In this sense, inorganic and organic nanoparticles were introduced to increase refractive index modulation and/or to reduce shrinkage. Liquid crystal polymer composites were added to the category of active photopolymer materials with an electrically switchable option.

This Special Issue focuses on polymeric and polymer nanocomposite materials for photonic applications and aims to provide new materials design, synthesis, and manufacture, and to explore the challenges posed by conventional and emerging applications, which will be shown in the 15 original articles that make up this Special Issue.

R. R. McLeod presents a study about noise gratings dynamics in holographic photopolymers [1]. The numerical approach is validated in the case of two-beam transmission holography in comparison to traditional harmonic series and rigorous coupled-mode approaches.

G. Panusa et al. demonstrate the fabrication of submicron polymer waveguides in PDMS by two-photon direct laser writing [2]. Their characterizations indicate the refractive index contrast 0.005 and 13 dB/cm of a relatively high transmission average loss, making for near achromaticity within the spectral band of 535–679 nm.

D. Bošnjaković et al. report on numerical simulation of holographic polymer-dispersed ferromagnetic liquid crystal [3]. The presented methodology provides a basis for designing new types of diffractive optical elements and their operation under in-plane magnetic fields.

A. M. Hussein et al. provide a detailed investigation of the film fabrication of polystyrene (PS) based polymer nanocomposites with a tuned refractive index and absorption edge [4]. They introduced SnTiO_3_ nanoparticles (NPs) to improve the properties. The prominent sharp and high-intensity peaks of the absorption edge resulted from the high incorporation of SnTiO_3_ NPs.

Y. Cao et al. demonstrate the development of Au nanoparticles-doped polymer optical switches based on the photothermal effect [5]. Compared with the based mode switch, the first-order mode switch gave lower power consumption and faster response time, despite large optical losses.

S-Y. Huang et al. show an electrically tunable nematic liquid crystal (NLC) diffraction grating with a periodic electrode structure [6]. The first order diffraction efficiency was electrically tuned, and the maximum first-order diffraction efficiency as high as ~12.5% was obtained with the applied external voltage of 5 V. By properly adjusting the applied voltage, a polarization state of a diffracted signal in this grating could be controlled electrically.

J. T. Sheridan et al. present a novel study using a thin drop cast layer of dry photosensitive materials to study the behaviors of wet photopolymer media using microscopic distances during the Self-Written Waveguide (SWW) process [7]. Three examples were studied: single-beam, counter-beam, and co-beam illumination. Their developed model can be used to more simply explore situations that would be difficult to implement experimentally.

J. Ma et al. present a biomedical application of Graphene Oxide Composite (GO-PEG-NH_2_), which is a multifunctional photothermal material for the selective recognition, capturing, and photothermal killing of bacteria over mammalian cells [8]. When the cells undergo bacterial infection, the higher negative charge density on the bacterial surface provides an inherent electrostatic driving force for GO-PEG-NH_2_, which can competitively bind to bacteria, but not to cells.

T. Lloret et al. analyze aberration and image quality of holographic lenses by means of a Hartmann–Shack wavefront sensor. Holographic lenses were stored in Biophotopol, a low-toxicity photopolymer [9]. They used different recording geometries (symmetric and asymmetric) and different reconstruction wavelengths (473 and 633 nm). One wavelength can be chosen indistinctly or it can be selected depending on the on-demand information.

K. Kiatkittipong et al. report an influence of titanium dioxide (TiO_2_) on antibacterial performance and show strength enhancement when it is blended with acrylonitrile-butadiene-styrene plastics (ABS) [10]. Under optimal conditions, an addition of silane could further improve TiO_2_ dispersion on ABS. This results in a decrease of bacterial survival by 75%.

Y. Liu et al. present an SiO_2_ nanoparticle (NP)-dispersed PQ/PMMA nanocomposite material (SiO_2_ NP-PQ/PMMA) with a high PQ doping concentration for holographic data storage [11]. They demonstrate the feasibility and potentiality of recording polarization-multiplexed holograms.

T-H. Chen et al. demonstrate the position-dependent emission wavelength of a colloidal crystal tunable laser with monodispersed silica particles [12]. Under position-dependent lattice constants, the wavelength of the lasing emission could be tuned from 604 nm to 594 nm.

O. Sakhno et al. explore a photosensitive nanocomposite doped with Au and Ag nanoparticles to fabricate submicrometer-scale grating structures by using a holographic method [13].

A. Beléndez et al. describe the generation of diffractive optical elements such as achromatic lenses, optical vortexes, and axicons with a spatial light modulator as a master LCoS and a photopolymer as an optical recording medium [14]. They also simulated the recording process by means of their diffusion model.

I. Pascual et al. analyze storage of volume holographic reflection gratings in low-toxicity photopolymer [15]. A maximum diffraction efficiency of 14.1% was reached after a curing process in 150 µm layer at a recording wavelength of 488 nm.

The scope of the works presented in this Special Issue offers a real insight into the progress made across a wide range of areas within the field of polymer nanocomposites and photopolymers. It is clear from this continued pursuit of knowledge that many exciting new applications and devices will be on the horizon in the near future.
